# Supplementary material for: The interactome of intact mitochondria by cross-linking mass spectrometry provides evidence for coexisting respiratory supercomplexes
Source: Mol Cell Proteomics. 2017 Dec 8;17(2):216–32. doi: 10.1074/mcp.RA117.000470 (PMC5795388; doi:10.1074/mcp.RA117.000470)
Supplement: Supplemental Data [file supp_RA117.000470_133922_0_supp_23978_fzffwf.zip › spectra_annotation/mito_spectra_annotation/380-1-8-1-21-1.pdf]

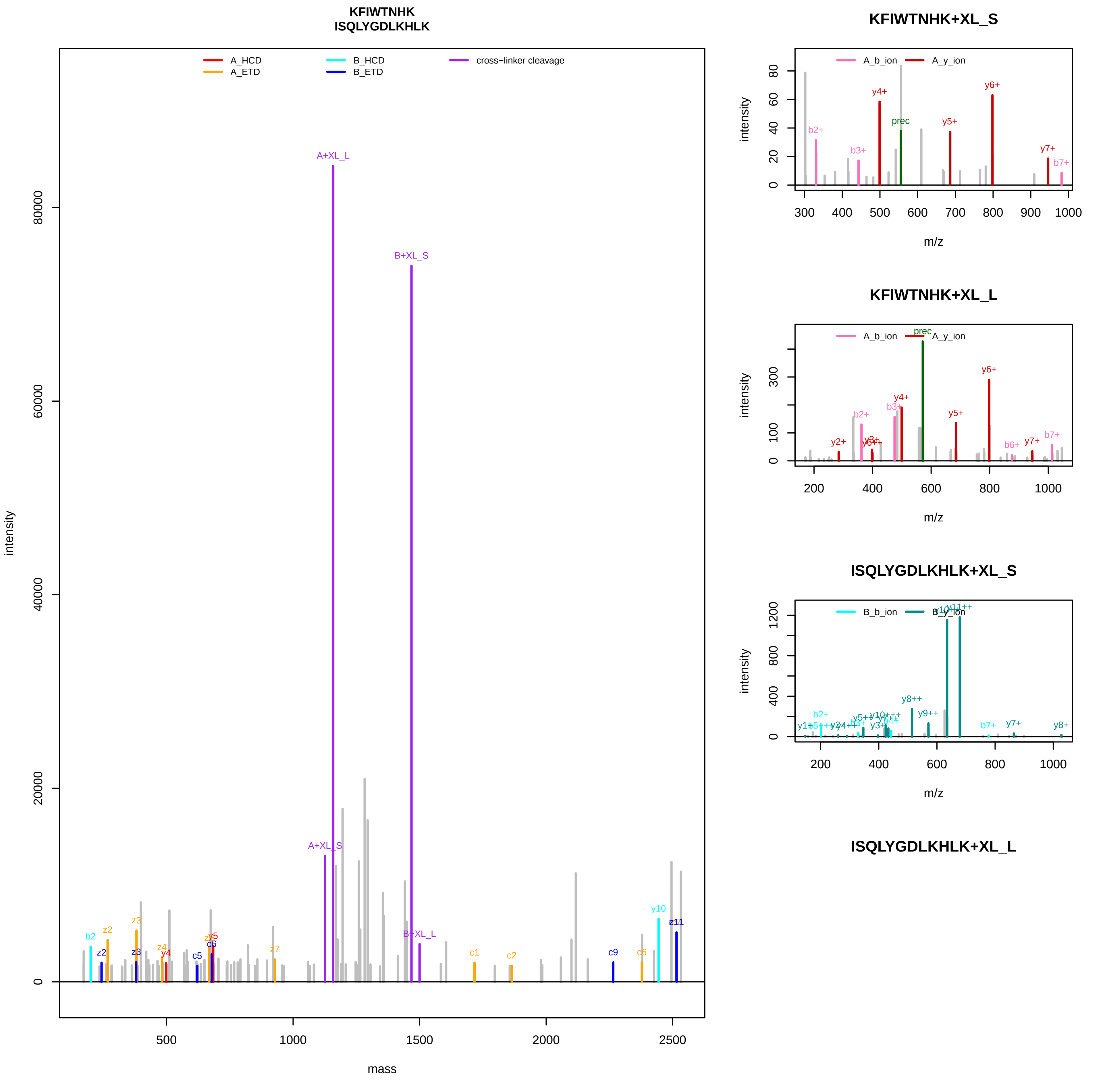
KFIWTNHK
ISQLYGDLKHLK
A_HCD
A_ETD
B_HCD
B_ETD
cross−linker cleavage
A+XL_L
B+XL_S
A+XL_S
B+XL_L
intensity
mass
0
20000
40000
60000
80000
500
1000
1500
2000
2500
b2
z2
z3
z4
y4
c5
c6
y5
z7
c1
c2
c9
c6
y10
z11
KFIWTNHK+XL_S
A_b_ion
A_y_ion
b2+
b3+
y4+
prec
y5+
y6+
y7+
b7+
m/z
KFIWTNHK+XL_L
y2+
b2+
b3+
y4+
y5+
y6+
b6+
y7+
b7+
ISQLYGDLKHLK+XL_S
B_b_ion
B_y_ion
b2+
y8++
y9++
y11++
b7+
y7+
y8+
ISQLYGDLKHLK+XL_L
